# Platelet-Activating Factor Receptor Ligands Protect Tumor Cells from Radiation-Induced Cell Death

**DOI:** 10.3389/fonc.2018.00010

**Published:** 2018-02-05

**Authors:** Ildefonso Alves da Silva-Junior, Barbara Dalmaso, Suellen Herbster, Ana Paula Lepique, Sonia Jancar

**Affiliations:** ^1^Instituto de Ciências Biomédicas, Universidade de São Paulo, São Paulo, Brazil

**Keywords:** platelet-activating factor receptor, prostaglandin E2, radiotherapy resistance, cervical cancer, head and neck squamous carcinoma

## Abstract

Irradiation generates oxidized phospholipids that activate platelet-activating factor receptor (PAFR) associated with pro-tumorigenic effects. Here, we investigated the involvement of PAFR in tumor cell survival after irradiation. Cervical cancer samples presented higher levels of PAF-receptor gene (PTAFR) when compared with normal cervical tissue. In cervical cancer patients submitted to radiotherapy (RT), the expression of PTAFR was significantly increased. Cervical cancer-derived cell lines (C33, SiHa, and HeLa) and squamous carcinoma cell lines (SCC90 and SCC78) express higher levels of PAFR mRNA and protein than immortalized keratinocytes. Gamma radiation increased PAFR expression and induced PAFR ligands and prostaglandin E2 (PGE2) in these tumor cells. The blocking of PAFR with the antagonist CV3938 before irradiation inhibited PGE2 and increased tumor cells death. Similarly, human carcinoma cells transfected with PAFR (KBP) were more resistant to radiation compared to those lacking the receptor (KBM). PGE2 production by irradiated KBP cells was also inhibited by CV3988. These results show that irradiation of carcinoma cells generates PAFR ligands that protect tumor cells from death and suggests that the combination of RT with a PAFR antagonist could be a promising strategy for cancer treatment.

## Introduction

Treatments based on cell death represent an effective way of controlling cancer growth locally, and radiotherapy (RT) represents the most effective postoperative treatment for cervical and head and neck squamous cell carcinoma (1, 2). However, they have an important drawback, which is the possibility of the exacerbated growth of tumor cells that survived treatment (a phenomenon known as tumor repopulation). Following cell death, homeostatic mechanisms are triggered within tissues, which favor the survival of surrounding cells. Apoptosis-induced proliferation is a physiological process that controls cell replacement in healthy or wounded tissue (3). However, in the tumor microenvironment, the apoptotic cells may promote undesirable proliferation of live radio-resistant tumor cells (4). Correa et al. (5) showed that the addition of apoptotic cells to a sub-tumorigenic dose of murine melanoma promoted tumor engraftment and growth. Later on, it was shown that this apoptosis-induced tumor growth was inhibited if mice were pretreated with antagonists of platelet-activating factor receptor (PAFR) (6).

Recently, using a mouse model to study irradiation-induced repopulation, we found that in both *in vivo* and *in vitro* experiments, irradiated TC-1 cells stimulated tumor cell proliferation in a PAFR-dependent manner. Irradiation also induced prostaglandin E2 (PGE2) production by a human carcinoma cell line transfected with PAFR (KBP) (7). Huang et al. (8) demonstrated that irradiated tumor cells undergoing apoptosis release factors that stimulate the growth of the surviving tumor cells by a mechanism dependent on the activation of caspase-3 and PGE2 secretion.

Both lipid mediators are released from membrane phospholipids after the activation of cytoplasmic phospholipase A2. The cleavage of phosphatidylcholine (GPC) generates arachidonic acid (AA) and lyso-GPC. The AA can be enzymatically converted to prostaglandins (9), while the lyso-GPC can be converted to PAF (alkyl-acyl-GPC) by PAF acetyl transferase (10). Besides the PAF generated by the enzymatic process, several oxidized phospholipids are generated by non-enzymatic processes (11). Irradiation generates reactive oxygen species, producing a wide range of oxidized phospholipids that also bind to PAFR (12, 13). These lipids exert their actions through G-protein-coupled receptors expressed in many cell types including some tumor cells. The expression of PAFR was shown in human melanoma SKmel-23, human breast cancer cells (MCF7, T-47D, and MDA-MB231), and EL4 cell lymphoma cell lines. The activation of PAFR in tumor cells was shown to increase proliferation (7, 13–15) and to induce the expression of antiapoptotic factors in B16F10 melanoma cells (16). Prostaglandin-inducible enzyme cyclooxygenase-2 is overexpressed in most solid tumors such as colorectal, liver, pancreatic, breast, and lung cancer (17–22), and the sustained biogenesis of PGE2 appears to play roles in tissue remodeling, angiogenesis, cancer cell survival, metastasis, and immune evasion (23–25). Thus, it seems that PAF and PGE2 have a pro-survival effect in tumor cells that express receptors for these mediators.

In the present study, we screened five carcinoma cell lines for the expression of PAFR, the effect of radiation on receptor expression, and the generation of PAF-like molecules and PGE2. Next, we investigated the effect of blocking PAFR or inhibiting prostaglandins in radiation-induced tumor cell survival.

## Materials and Methods

### Expression Datasets

Gene Expression Omnibus (GEO^1^) is an open database providing gene expression data and clinical data information. We retrieved cervical cancer datasets from the GSE9750 and GSE3578 and compared PAF-receptor gene expression (PTAFR) among the groups. Data were analyzed by non-parametric Mann–Whitney test to compare groups from GSE9750 and Wilcoxon test to compare paired samples from GSE3578. All statistical tests were two-sided. Datasets were analyzed for outliers using https://graphpad.com/quickcalcs/grubbs1/. A *P*-value less than 0.05 was considered to be statistically significant.

### Cell Lines and Culture Conditions

Cervical cancer-derived cell lines (C33, SiHa, and HeLa) and squamous cell carcinoma cells lines (SCC78 and SCC90) were kindly donated by Professor Luisa Lina Villa (ICESP, Sao Paulo, SP, Brazil). HeLa, SiHa, and SCC90 cell lines are positive for HPV, while C33 and SCC78 cell lines are negative for HPV but have mutated p53. HaCaT, an immortalized keratinocyte used as control, was purchased from the American Type Culture Collection (Manassas, VA, USA). Cells were maintained in RPMI 1640, supplemented with 10% FBS (fetal bovine serum), penicillin (100 U/mL), and streptomycin (100 µg/mL). In addition, we obtained a PAFR-negative human epithelial cell line (KBM) and PAF-receptor-positive (KBP) cells from Dr. J. B. Travers (Department of Dermatology, Indiana University School of Medicine, Indianapolis, IN, USA). These cell lines were cultured in Dulbecco’s Modified Eagle’s Medium (DMEM, GIBCO, OK, USA) supplemented with 10% FCS, penicillin (100 U/mL), and streptomycin (100 µg/mL). Cells were regularly tested for Mycoplasma and were free of this contamination. All cell cultures were incubated at 37°C under a humidified atmosphere of air containing 5% CO_2_.

### *In Vitro* Irradiation of Tumor Cells

Cell lines were grown on 10-cm dishes to 80–90% confluence and washed three times with pre-warmed (37°C) PBS and then cultured in RPMI medium containing 2% of fetal bovine serum (FBS) for short-term cultures (4 h) or 10% of FBS for long-term cultures (72 h) as indicated in figure legends. Tumor cells were irradiated with multiple doses of gamma radiation (Gy). Cell irradiation studies were conducted using an IBL 136 cell and animal gamma radiator machine (Compagnie Oris Industrie, France). Settings for the machine were as follows: *d* = 33 c and dose rate of 251.7 cGy/min. In some experiments, the PAFR antagonist CV3988 (Enzo Life Sciences, Farmingdale, NY, USA) or the vehicle control dimethyl sulfoxide of 0.5% was pre-incubated for 30 min before irradiation.

### Measurement of PAFR Ligands after Irradiation

Tumor cells and HaCaT cells (10^7^ mL) were plated in 10-cm dishes and incubated overnight in RPMI supplemented with 10% FBS. Following incubation, the culture medium was replaced with 2 mL of pre-warmed (37°C) Hanks Balanced Salt Solution supplemented with 10 mg/mL fatty acid-free bovine serum albumin and 2 µM Pefabloc, a serine hydrolase inhibitor that blocks PAF degradation (Sigma-Aldrich, St. Louis, MO, USA). The cells were then irradiated with 4–8-Gy doses following a 1-h incubation. The culture was quenched by the addition of 2 mL of ice-cold methanol followed by methylene chloride, and lipids were extracted, as previously described [(26–28), p. 50]. The presence of PAFR ligands in lipid extracts was determined by the ability of these extracts to induce interleukin-8 (IL-8) production in KBP cells, as previously described (29). Briefly, 2 × 10^5^ KBP cells were plated in 12-well plates and cultured overnight in DMEM containing 10% FBS. The cells were then washed with PBS and incubated with FBS-free DMEM. The cells were stimulated with the lipid extracts from irradiated cells, and after 5 h, the supernatants were collected for IL-8 measurement. The concentration of IL-8 induced by the lipid extracts was compared with that induced by the stable PAFR agonist cPAF (1-hexadecyl-2-*N*-methylcarbamoyl glycerolphospho-choline). The concentration of IL-8 in the supernatants was measured using BD OptEIA™ ELISA sets (BD Biosciences), according to the manufacturer’s instructions.

### RNA Analysis for PAFR Expression

Platelet-activating factor receptor expression was analyzed by quantitative reverse transcription polymerase chain reaction using RNA obtained from HaCaT and tumor cells. Total RNA was isolated using TRIzol Reagent (Life Technologies). For real-time RT-PCR, cDNA was synthesized using the RevertAid™ First Strand cDNA Synthesis Kit (Fermentas Life Sciences, Ontario, CA, USA), according to the manufacturer’s instructions. PCR Master Mix (Power SyBr^®^ Green, Applied Biosystems, Warrington, UK) containing the specific primers was then added. Human PAF receptor forward primer: GGG GAC CCC CAT CTG CCT CA and reverse GCG GGC AAA GAC CCA CAG CA; GAPDH forward primer: GAG TCA ACG GAT TTG GTC GT and reverse primer: TTG ATT TTG GAG GGA TCT CG. Real-time PCR was performed using the Mx3005PTM Real-Time PCR System (Stratagene). Relative gene expression was calculated using the 2^−ΔΔCT^ method, as previously described (30). Results are presented as a fold increase relative to non-irradiated cells.

### Flow Cytometry for PAFR Expression

Tumor cells and HaCaT cells (10^7^ mL) were incubated overnight in a medium supplemented with 10% FBS. Following incubation, the culture medium was replaced with 2 mL of ice-cold phosphate buffer solution (PBS), and the cells were removed with a rubber policeman cell scraper and harvested by centrifugation at 250 × *g* for 5 min. Following centrifugation, the cell pellet was washed and resuspended in the staining buffer (PBS, FCS 1%, and sodium azide 0.1%) containing the primary antibody (rabbit IgG to PAFR 1:100 dilution in staining buffer; Cayman Chemical, Ann Arbor, MI, USA). Following a 30-min incubation, the cells were washed and resuspended in staining buffer containing Alexa Fluor 647-goat anti-rabbit IgG secondary antibody (1:100 dilution in staining buffer; Invitrogen Life Technologies, Carlsbad, CA, USA). Cells incubated with secondary antibody only were used to control for background fluorescence. The expression of the PAFR was analyzed by flow cytometry. During data acquisition, the doublets were excluded using gates in FSC-A vs. FSC-H.

### ELISA Measurement of PGE2

To measure PGE2 secretion by tumor cells, 10^6^ cells per well were plated in 10-cm dishes and incubated overnight. The cells were treated with the prostaglandin inhibitor indomethacin (Sigma-Aldrich, St. Louis, MO, USA) or the PAFR antagonist CV3988 for 30 min prior to Gy treatment. The supernatants from the cells were collected 1 h after cell irradiation, and PGE2 levels in the supernatants were measured by ELISA (Cayman Chemical, Minneapolis, MN, USA).

### Cell Death Assay

The carcinoma cell lines (2 × 10^5^) treated with different doses of radiation (4 or 8 Gy) were incubated in RPMI 10% FBS at 37°C with 5% CO_2_ for 72 hs. Cell death was assessed by labeling the tumor cells with annexin V FITC (5 µL per sample, BD Biosciences) and propionate iodide (PI) (10 µL per test, BD Biosciences) for 15 min and then analyzed by flow cytometry using BD FACS Calibur (BD Biosciences) and FlowJo Version 5.0 software (Ashland, OR, USA). The cells positive for both annexin V and PI were considered as undergoing cell death.

### Statistical Analysis

Data are represented as mean ± SD unless otherwise indicated and were analyzed using the Prism 5.0 statistical program (GraphPad Software, San Diego, CA, USA). Comparisons among groups were performed by analysis of variance followed by the Bonferroni multiple comparison test. A two-sided *P* < 0.05 was considered to be statistically significant. Each experiment was repeated at least three times.

## Results

### Irradiation of Tumor Cells Increases PAFR Expression and Induces PAF-Like Lipids and PGE2

**Figure 1:**
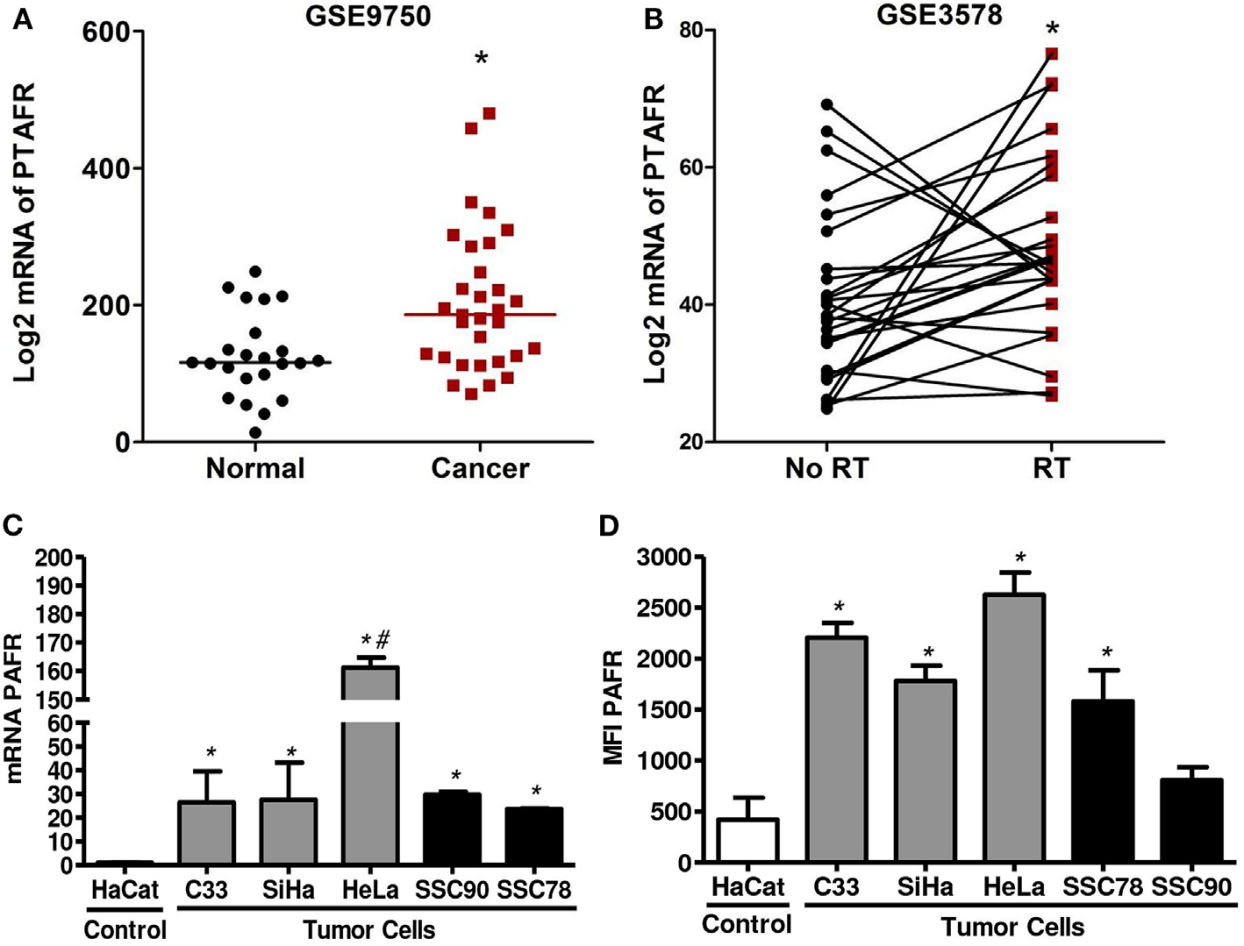
Platelet-activating factor receptor (PAFR) expression was significantly upregulated in cervical invasive carcinomas and cervical cancer-derived cell lines. **(A)** Analysis PAFR (PTAFR) was increased in invasive carcinomas when compared with normal cervical tissues (GSE9750; *p* = 0.0044). **(B)** The PTAFR expression was upregulated in cervical tumors from patients who underwent radiotherapy when compared with biopsies taken before treatment (GSE3578; *p* = 0.0208). These two cervical cancer datasets were retrieved from GEO web database. **(C)** PAFR mRNA expression was analyzed by RT-qPCR in cervical tumor cells (C33, SiHa, and HeLa), head and neck squamous cell carcinoma lineage (SCC90 and SCC78), and a control keratinocyte (HaCaT). Data are shown as the mean ± SD of the comparative delta-CT related to HaCaT of three independent experiments. **(D)** Median fluorescence intensity (MFI) of PAFR protein expression in the cell membrane. Data were obtained from three independent experiments. *P < 0.05 comparing the tumor cells with the control keratinocyte HaCaT.

We first analyzed PAFR (PTAFR) expression in two cervical cancer datasets retrieved from GEO web database.^2^ We observed that PTAFR expression was upregulated in invasive carcinomas (*n* = 33) when compared with age-matched normal cervical tissues from hysterectomy specimens (*n* = 19) (GSE9750, Figure 1A) (31). In the second series (GSE3578), we found that the mRNA level of PTAFR was increased in cervical tumors (*n* = 26) after the patients underwent RT treatment protocol (Figure 1B). Biopsies were taken before and 1 week after the start of therapy. For the after-treatment biopsy, the patients had received 9 Gy of whole pelvic irradiation, part of a 30.6-Gy RT protocol to the whole pelvis, plus an additional dose to parametria with central shielding to complete 50.6-Gy protocol treatment, along with ^192^Ir high-dose rate intracavitary [described in Ref. (32)].

To improve this observation, we also evaluate the expression of PAFR in three cervical carcinoma cell lines (C33, SiHa, and HeLa) and in two squamous carcinoma cell lines (SCC90 and SCC78) compared to an immortalized keratinocyte (HaCaT). Our results showed that all carcinoma tumor cell lines express higher amounts of mRNA for PAFR than the control HaCaT cell line (Figure 1C). We also evaluated membrane PAFR expression by flow cytometry and observed that C33, SiHa, HeLa, and SCC78 cell lines express higher amounts of the receptor in relation to HaCaT cells (Figure 1D).

**Figure 2:**
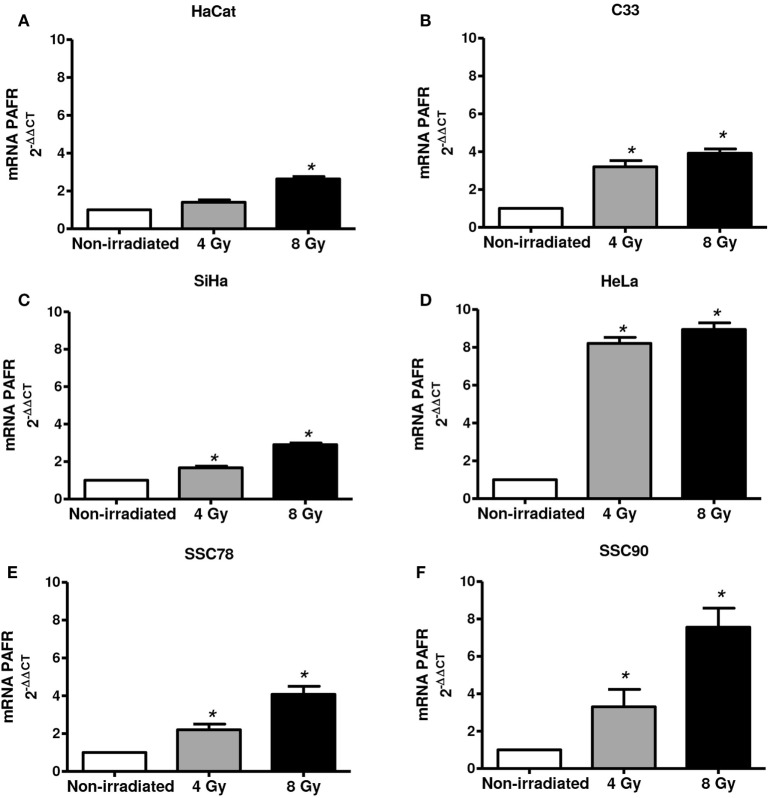
Irradiation of tumor cells increases platelet-activating factor receptor (PAFR) expression. Immortalized keratinocytes HaCaT **(A)**, human cervical carcinoma cell lines C33 **(B)**, SiHa **(C)**, and HeLa **(D)** and head and neck squamous cell carcinoma lineage SCC78 **(E)** and SCC90 **(F)** were cultured in RPMI 2% fetal bovine serum at 37°C, and 5% CO_2_ was irradiated with 4 or 8 Gy. After 6 h, PAFR mRNA expression was analyzed by RT-qPCR. Data are shown as the mean ± SD of three independent experiments. *P < 0.05 comparing irradiated with non-irradiated groups.

**Figure 3:**
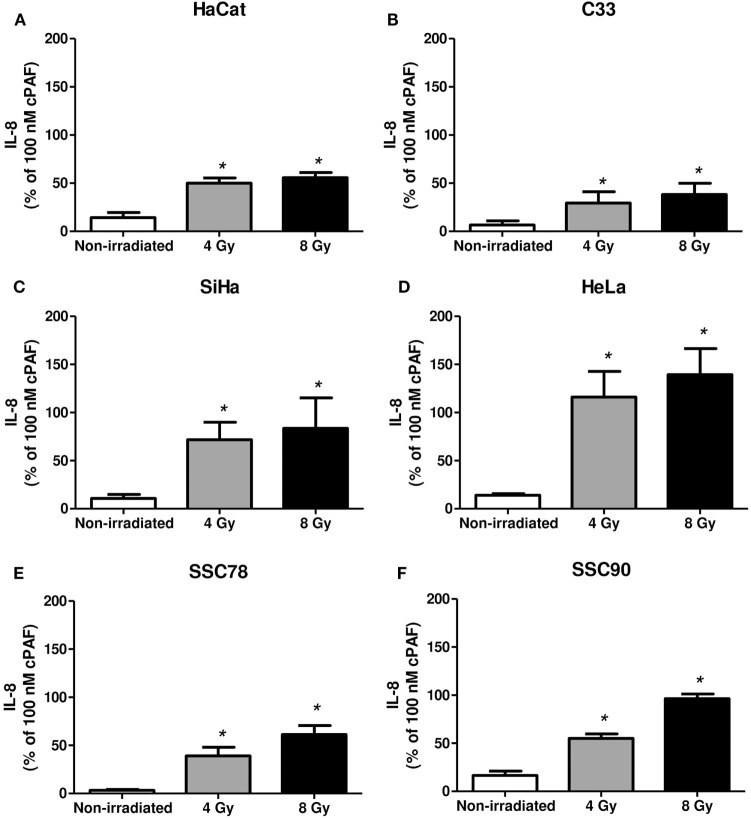
Irradiation of tumor cells induces platelet-activating factor receptor (PAFR) ligands production. Immortalized keratinocytes HaCaT **(A)**, human cervical carcinoma cell lines C33 **(B)**, SiHa **(C)**, and HeLa **(D)** and head and neck squamous cell carcinoma lineage SCC78 **(E)** and SCC90 **(F)** were cultured in RPMI 2% fetal bovine serum at 37°C, and 5% CO_2_ was irradiated with 4 or 8 Gy. Lipid extracts were obtained after 1 h following treatment and then added to KBP cells. After 6 h, interleukin-8 (IL-8) was quantified as a measure of PAFR agonistic activity. As control, PAF (100 nM) was added to KBP cells. Results are expressed as the percentage of IL-8 relative to amounts induced by cPAF. Mean ± SD of six experiments made in duplicate. *P < 0.05, comparing irradiated with non-irradiated control.

Next, we assessed whether irradiation would affect PAFR expression in these cell lines. As shown in Figure 2, irradiation of all cell lines *in vitro* (4 or 8 Gy) increased PAFR mRNA levels. The lipid extracts from the irradiated cultures obtained as described in Section “Materials and Methods” were then assayed for their capacity to activate PAFR. For this purpose, we developed an assay with carcinoma cells transfected with PAFR (KBP) which secrete (IL-8) in response to receptor activation (33, 34). A concentration response curve to the PAFR agonist methylcarbamyl PAF was performed and used to calculate the concentration of PAFR-like lipids present in the supernatants of irradiated cells. This is a very useful assay as it allows the detection of the collection of PAFR ligands generated by irradiation. Figure 3 shows that irradiation induced the secretion of PAFR ligands by all carcinoma cells investigated. These results indicate that PAF-like molecules are produced by radiation and enhance PAFR expression in tumor cells.

**Figure 4:**
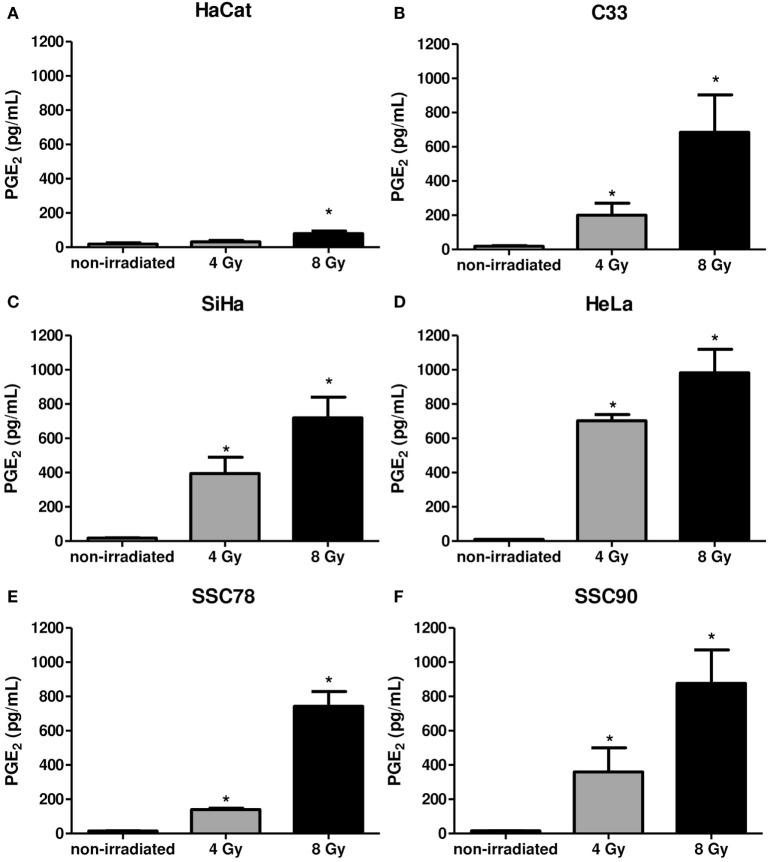
Irradiation of tumor cells induces prostaglandin E2 (PGE2) production. Immortalized keratinocytes HaCaT **(A)**, human cervical carcinoma cell lines C33 **(B)**, SiHa **(C)**, and HeLa **(D)** and head and neck squamous cell carcinoma lineage SCC78 **(E)** and SCC90 **(F)** were cultured in RPMI 2% fetal bovine serum at 37°C, and 5% CO_2_ was irradiated with 4 or 8 Gy. After 6 hs, the PGE2 production was evaluated in the supernatants. Data are presented as mean ± SE (standard error) of three independent experiments. *P < 0.05, comparing irradiated with non-irradiated control.

Supernatants of the carcinoma cells collected 6 h after irradiation were measured for PGE2 levels. Figure 4 shows the irradiation-induced dose-dependent generation of PGE2 by all tumor cells. Taken together, these results show that in the carcinoma cell lines screened, Gy increased PAFR expression and induced PAF-like molecules and PGE2 generation.

### PAFR Activation in Tumor Cells Protects Them from Radiation-Induced Death

**Figure 5:**
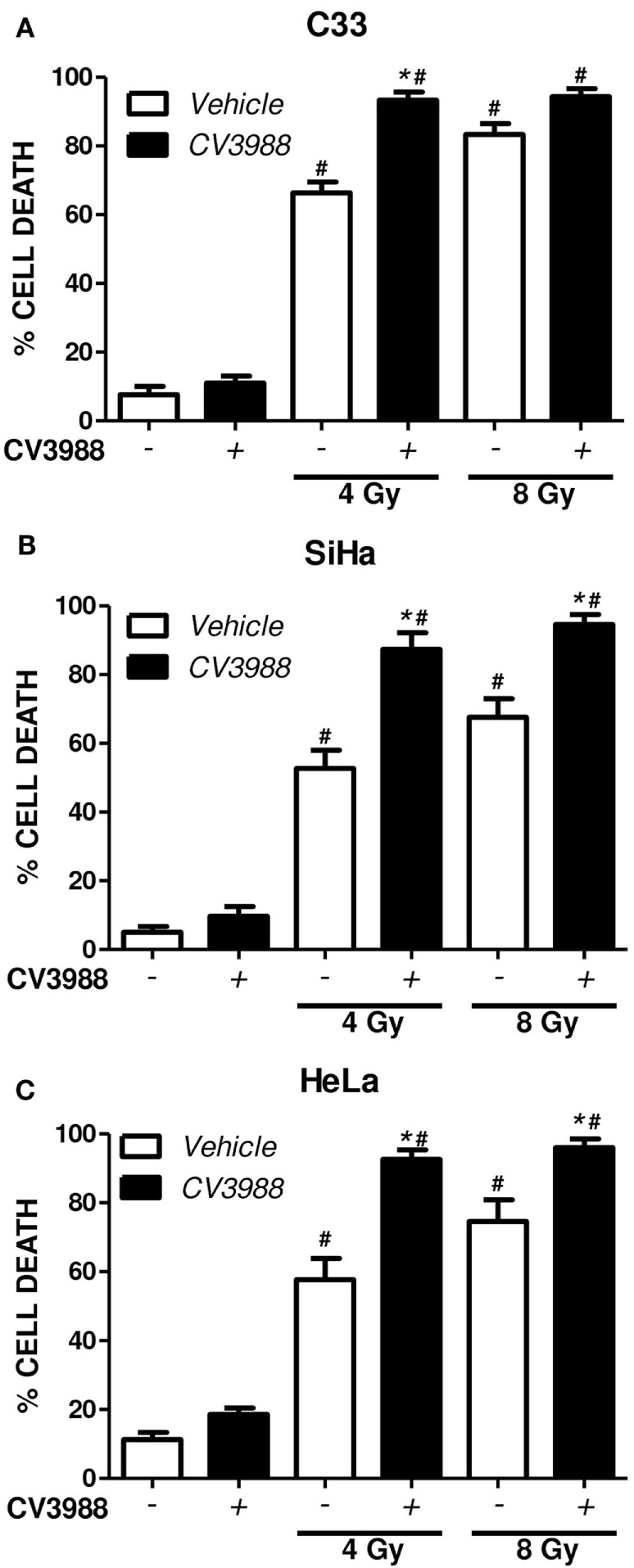
Platelet-activating factor receptor (PAFR) antagonist enhances cell death induced by radiotherapy. C33 **(A)**, SiHa **(B)**, and HeLa **(C)** cervical carcinoma cell lines were treated (black bars) or not (white bars) with PAFR antagonist (10 µM CV3938) 30 min before irradiation with 4 or 8 Gy. After 3 days, the cultures were trypsinized and stained with annexin V FITC and propidium iodide (PI). Data are shown as the mean ± SE (standard error) of the percentage of cells positive for PI and annexin V. ^#^*P* < 0.05 irradiated vs. non-irradiated and *P < 0.05 treated vs. not treated with PAFR antagonist CV3938. *n* = 3 independent experiments performed in duplicate.

To investigate if PAFR-mediated mechanisms are involved in tumor resistance to radiation, we pretreated the C33, SiHa, and HeLa tumor cells with the PAFR antagonist CV3938 before irradiation (4 or 8 Gy) of the cells and assessed cell death after 3 days. Death was assessed by labeling the tumor cells treated with annexin V and PI and analyzing by flow cytometry. Gy-induced tumor cell death and blockade of PAFR with CV3938 further increased cell death (Figure 5).

**Figure 6:**
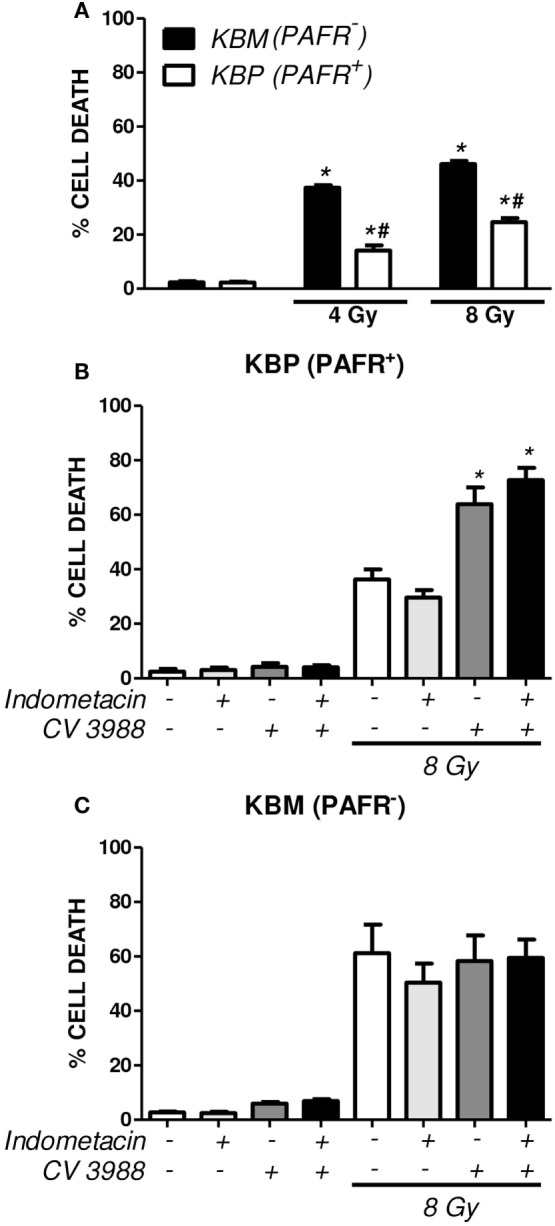
Platelet-activating factor receptor (PAFR) expression protects carcinoma cells from radiation-induced cell death. Human carcinoma cells transfected with PAFR (KBP) and negative for PAFR (KBM) were irradiated with 4 or 8 Gy and incubated for 3 days. **(A)** Cells were stained with annexin V FITC and propidium iodide (PI), and the percentage of death of cells (annexin/PI-positive cells) was determined (*P < 0.05 KBM vs. KBP cells; ^#^*P* < 0.05 irradiated vs. non-irradiated). A similar protocol was applied in **(B)** KBP and **(C)** KBM cells treated or not with the COX-inhibitor indomethacin (15 µM) or PAFR antagonist (10 µM CV3938) 30 min before irradiation. Results are shown as percentage of apoptotic cells (*P < 0.05).

**Figure 7:**
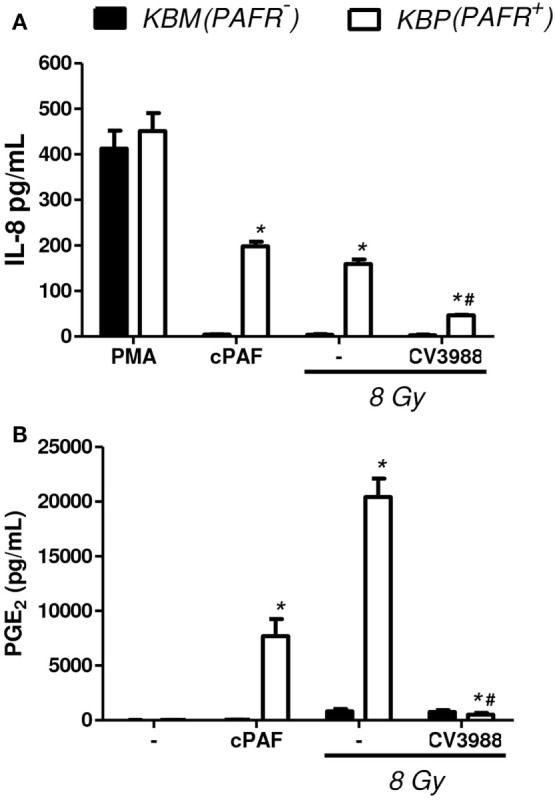
Irradiation of tumor cells transfected with PAFR: the production of PAFR agonists and PGE2. Human carcinoma cells transfected with PAFR (KBP) and negative for PAFR (KBM) were cultured in high density (10^6^ cells/mL) and were treated or not with gamma irradiation (8 Gy). **(A)** Interleukin-8 (IL-8) concentration in cell supernatants 6 h after irradiation was quantified by ELISA as a measure of molecules with agonist activity on the PAF receptor. As control, cPAF (100 nM) or phorbol 12-myristate 13-acetate (PMA) (1,000 nM) was added to cells. **(B)** PGE2 concentration in cell supernatants 1 h after irradiation. Results are expressed as the median ± SE (standard error) of three experiments made in duplicate. *P* < 0.05 KBM vs. KBP cells; ^#^*P* < 0.05 treated vs. not treated with PAFR antagonist CV3938.

In another experimental approach, the human carcinoma cell line (KBM) that does not express PAFR and the same cells transfected with the PAFR (KBP) were irradiated, and the cytotoxic effect of radiation was measured. After 72 h of exposure to 4 or 8 Gy, the PAFR^+^ cells survived more than the PAFR^−^ cells (Figure 6A), confirming the protective effect of PAF. Furthermore, the KBP cells were treated with a PAFR antagonist or a prostaglandin synthesis inhibitor (indomethacin) before irradiation. Figure 6B shows that the treatment of KBP cells with CV3988 significantly increased radiation-induced cell death, whereas indomethacin did not affect cell survival (Figure 6B). At the dose of indomethacin used, it abolished the PGE2 in the supernatants of irradiated KBP cells (data not shown). In PAFR^−^ cells (Figure 6C), radiation increased cell death, and this was not affected by the treatments. In Figure 7A, we can see that KBP cells irradiated with 8 Gy produced an amount of PAFR ligands equivalent to 92% of 100 nM of cPAF. KBM did not respond to PAF or to irradiation, although they did respond to phorbol 12-myristate 13-acetate stimulation. Irradiated KBP cells produced PGE2, and this was almost abolished by CV3988 (Figure 7B). These results suggest that PAFR ligands produced during irradiation are more relevant for the radio resistance of tumor cells than prostaglandins.

## Discussion

Previous studies have shown that PAFR activation results in accelerated proliferation, angiogenesis, and metastasis in different models of melanoma, breast, ovarian, and prostate tumors (9, 35, 36). The association of PAFR antagonists with chemotherapy agents leads to increased tumor cell death and improved treatment effectiveness (14, 37–39). We have recently found similar effects with the association of PAFR antagonists and RT (7, 13, 40). The present study provides additional support for the functional significance of the PAF/PAFR system in tumor RT. Our results show that PTAFR expression was upregulated in cervical cancer clinical samples when compared to normal cervical tissue. Also, PTAFR mRNA level was increased in cervical tumors after RT treatment. In the carcinoma cell lines screened, Gy increased PTARF expression and induced PGE2- and PAF-like lipids. The activation of PAFR by these lipids protects tumor cells from radiation-induced cell death. Moreover, it seems that the PAFR ligands are more relevant for tumor cell’s radio resistance than prostaglandins.

The expression of PAFR is elevated in different types of cancer, such as human hepatocellular carcinoma (41), gastric adenocarcinoma (42), and by several human tumor lineages [e.g., Kaposi’s sarcoma cells (43), endometrial cancer cell line HEC-1A (44), epidermoid carcinoma (A431 cells) (45), stomach cancer cell line JR-St (46), and N1E-115 neuroblastoma cells (47)]. Our data show that PAFR is also overexpressed in cervical and head and neck squamous cell carcinoma lineages, which suggest that it may have a role in the pathogenesis and progression of these types of tumors.

Previous studies from our laboratory showed that the stimulation of a cell line positive for PAFR (KBP) with cPAF increased cell proliferation; however, this did not occur in PAFR^-^ (KBM) control cells (7). Also, the activation of PAFR induces the phosphorylation of ERK and p38 MAP kinase in KBP cells, which stimulates cell proliferation (48). PAF seems to have a role in tumor growth in normal conditions, since transgenic mice overexpressing PAFR exhibited increased local cell growth in the skin, characterized by dermal and epidermal hyperthickening with dermal melanosis, and in some aged mice, spontaneous development of melanocytic tumors (48) occurred.

Some previous studies have also linked the PAF/PAFR axis with cellular responses to stress and cellular damage. Different stimuli including cytokines (49), cigarette smoke (27), ultraviolet B (28), and gamma irradiation (13) resulted in significant levels of PAF synthesis by tumors cells. The approach used in this study was to induce irradiation stress in tumors cells after the inhibition of PAFR to enhance the sensitivity of cancer cells toward radiation. The present study was performed with a gamma irradiator at various radiation doses (4 and 8 Gy). It is well established that radiation sensitivity mechanisms in human cancers are determined by intracellular factors (18). Conversely, in this study, we demonstrate that upon different doses of radiation, cervical tumor cells and head and neck carcinoma cells respond by increasing PAFR expression and producing a great deal of PAFR ligands that could affect cell survival. Previous work from Seo et al. (16) showed that PAFR activation induces the upregulation of antiapoptotic gene products, such as Bcl-2, thus attenuating the cytotoxic effect of chemotherapeutic agents. Like this, specific PAFR antagonists may have a potential effect in blocking protective tumor responses and potentiating RT.

In fact, the simultaneous association of PAFR antagonists with low doses of radiation increased cell death induced by cell damage, demonstrating the protective effect of the PAFR on tumor cell lines. In addition, the irradiation-induced PAF ligands signaling cascade alone was sufficient to induce PGE production, which in turn is highly associated with the activation of antiapoptotic pathways (50, 51) and the phenomenon of tumor repopulation induced by RT (8, 52, 53).

To support our hypothesis that PAFR can protect cells from irradiation-induced apoptosis *via* PGE production, we demonstrate that irradiated PAFR^+^ tumor cells show greater resistance to radiation-induced cell death than PAFR^-^ tumor cells. This resistance was suppressed by treatment with the CV3988 antagonist. An additional effect on the loss of radio resistance to cell death was observed by treatment with the prostaglandin inhibitor indomethacin. This evidence supports our conclusion that PAFR expression is positively correlated with cell survival.

## Conclusion

Together, our data suggest that (1) radiation induces PAFR expression and the corresponding ligands (PAF-like molecules) and (2) the inhibition of PAFR selectively enhances the sensitivity of cervical carcinoma and squamous carcinoma cells to radiation treatment. These results are of potential interest as they widen the field of investigation into the roles of PAFR and support the concept that PAFR antagonists may be useful for adjuvant therapy, improving the efficacy of RT.

## Author Contributions

IA and SJ participated in the conception and design of the study; IA, BD, and SH performed acquisition of data, analysis, and interpretation of data; and IA, SJ, and AL wrote the manuscript.

## Conflict of Interest Statement

The authors declare that the research was conducted in the absence of any commercial or financial relationships that could be construed as a potential conflict of interest.

## Footnotes

^1^http://www.ncbi.nlm.nih.gov/geo.

^2^https://www.ncbi.nlm.nih.gov/gds.
